# Gene Expression Profile in the Liver of BALB/c Mice Infected with *Fasciola hepatica*

**DOI:** 10.1371/journal.pone.0134910

**Published:** 2015-08-06

**Authors:** Jose Rojas-Caraballo, Julio López-Abán, Pedro Fernández-Soto, Belén Vicente, Francisco Collía, Antonio Muro

**Affiliations:** 1 Instituto de Investigación Biomédica de Salamanca—Centro de Investigación de Enfermedades Tropicales de la Universidad de Salamanca (IBSAL-CIETUS), Facultad de Farmacia, Universidad de Salamanca, Salamanca, Castilla y León, España; 2 Departamento de Anatomía e Histología Humanas, Facultad de Medicina, Universidad de Salamanca, Salamanca, Castilla y León, España; Centro de Investigacion y de Estudios Avanzados del Instituto Politecnico Nacional, MEXICO

## Abstract

**Background:**

*Fasciola hepatica* infection still remains one of the helminthic neglected tropical diseases (NTDs). It has a huge worldwide distribution, affecting mainly cattle and, sometimes, human beings. In addition to data reported about the immunological response induced by helminthic infections and that induced by *Fasciola hepatica*, little is known about the gene expression profile in its organ target, the liver, which is where adult worms are established and live for long periods of time, causing its characteristic pathology. In the present work, we study both the early and late gene expression profiles in the livers of mice infected with *F*. *hepatica* metacercariae using a microarray-based methodology.

**Methodology:**

A total of 9 female-6-week-old BALB/c mice (Charles River Laboratories, Barcelona, Spain) weighing 20 to 35 g were used for the experiments. Two groups of BALB/c mice were orally infected with seven *F*. *hepatica* metacercariae, and the other group remained untreated and served as a control. Mice were humanely euthanized and necropsied for liver recovery, histological assessment of hepatic damage, RNA isolation, microarray design and gene expression analysis on the day of infection (t0), seven days post-infection (t7) and twenty-one days post-infection (t21).

**Results:**

We found that *F*. *hepatica* infection induces the differential expression of 128 genes in the liver in the early stage of infection and 308 genes in the late stage, and most of them are up-regulated. The Ingenuity Pathway Analysis revealed significant changes in the pathways related to metabolism, biosynthesis and signaling as well as genes implicated in inducing liver-toxicity, injury and death.

**Conclusion:**

The present study provides us insights at the molecular level about the underlying mechanisms used by *F*. *hepatica*, leading to liver damage and its subsequent pathophysiology. The expression pattern obtained here could also be used to explain the lack of association between infection with *F*. *hepatica* and cholangiocarcinoma. However, more studies should be performed to confirm this hypothesis.

## Introduction

Fasciolosis is considered one of the most widespread foodborne and waterborne trematodiasis worldwide. It is caused by flukes of the genus *Fasciola*, being *Fasciola hepatica* and *Fasciola gigantica* the both species responsible for the disease, which mainly affects ruminants but can also affect humans, causing important economic losses in developing countries (estimated at over 3 billion dollars per year) [1, 2]. Human fasciolosis is distributed in countries from South America (Andean countries), the Middle East (Egypt and Iran), the Asian continent and sub-Saharan Africa, where it is estimated that up to 17 million people are infected, and close to 170 million people live in areas with a high risk of infection [3–6]. *F*. *hepatica* infection occurs in the definitive host (animal and human) by a series of successful successive events, which determines the acute and chronic phases of fasciolosis, as follows: *i)* oral ingestion of some aquatic plants or water contaminated with the larval stages of *F*. *hepatica*, the metacercariae; *ii)* excystment in the duodenum and migration through the intestinal wall, peritoneal cavity and Glisson’s capsule; and *iii)* entry into the liver parenchyma and establishment in the biliary ducts [7].

The acute phase is characterized by the process of migration, which causes liver and abdominal peritoneum damage as the parasites move across these tissues. Some characteristic frequent symptoms at this stage include fever, abdominal pain and gastrointestinal disorders, but some respiratory-associated clinical disorders could appear, such as cough, dyspnea and chest pain [7, 8]. The chronic phase of fasciolosis is typically characterized by the establishment and maturation of *F*. *hepatica* worms into the biliary ducts of its definitive host. This stage of infection could last from several months to years. Chronic *F*. *hepatica* infection causes hepatomegaly, gallbladder and biliary duct thickening and dilatation, leading to cholangitis, cholecystitis and, usually, the obstruction of the biliary ducts, causing hepatic dysfunction in some cases [7–9].

In the present study we are interested in testing the gene expression profile at both early (7 days, the parasite is still considered newly excysted juvenile, the macroscopic lesions in the liver starts to be evident and the immunomodulation induced by the parasite begins) and late (21 days, which corresponds to parasite migration through the hepatic parenchyma and subsequent establishment of mature adults in the liver) stage of infection. However, literature reports concerning precise chronobiology of *F*. *hepatica* development and migration in mice is still scarce and it could be variable from one mouse to another and also from one strain to another. There are previous reports in the literature describing some aspects from the chronobiology of *F*. *hepatica* in mice [10, 11].

For many years, several efforts have been made to control fasciolosis-causing infection. One of the most appealing strategies for such prevention is the massive administration of triclabendazole, which is the only chemotherapeutic agent that is highly effective against both immature and mature liver flukes. To date, it is the drug of choice for treating fasciolosis in both animals and humans [12, 13]. However, this control strategy has resulted in increasing triclabendazole resistance, leading to the search of new control strategies [14–16]. The development of an effective vaccine represents one strategy for reducing the risk of infection. However, there are no commercial vaccines available even though significant progress has been made in the attempts to develop effective vaccines against infections that are caused by parasites. To date, only a few vaccines have been successfully tested against helminthic infection in animals [17–19].

Although the pathophysiology and immune response induced by *F*. *hepatica* infection has been well characterized, little is known about the precise cellular and molecular mechanisms that lead to such pathology. A deeper understanding of the molecular mechanisms by which *F*. *hepatica* infection occurs and induces liver pathology has become a priority for not only selecting new drug-alternatives for treatment but also in the search for new vaccine candidates. In the present study, we used the mouse as experimental model due to its high susceptibility to acquire the infection to investigate the gene expression profile in the livers of mice that were orally infected with *F*. *hepatica* metacercariae at different times of infection using a microarray-based methodology to identify the set of genes involved in causing liver damage.

## Materials and Methods

### Ethics Statement

The animal procedures in this study complied with the Spanish (Real Decreto RD53/2013) and European Union (European Directive 2010/63/CE) guidelines regarding animal experimentation for the protection and humane use of laboratory animals. The University of Salamanca’s accredited Animal Experimentation Facilities (Registration number PAE/SA/001) were used for such procedures. The University of Salamanca’s Ethics Committee also approved the procedures that were used in this study (Permit Number: 8402). The animals’ health status was monitored throughout the experiments by a health surveillance program according to Federation of European Laboratory Animal Science Associations (FELASA) guidelines. All efforts were made to minimize suffering.

### Experimental design, mice and parasites

A time-course experiment to investigate the gene expression profile over time in the liver of mice infected with *F*. *hepatica* metacercariae for three-different experimental groups was performed. We are interested in testing the gene expression profile at both early (7 days) and late (21 days) stage of infection. We also find interesting to investigate the immunological changes and the damage induced by *F*. *hepatica* during its migration through the host’s tissues by studying the transcriptome profile at this stages of infection.

Female, 6 week-old BALB/c mice (Charles River Laboratories, Barcelona, Spain) weighing 20 to 35 g were used for the experiments. Animals were maintained in the University of Salamanca’s animal care facility and kept in plastic boxes with food and water *ad libitum*. The animals were used in experiments after a period of 7 days of adaptation in captivity, with regular 12 h light–dark periods at 20°C (see S1 File: NC3Rs ARRIVE Guidelines Checklist). *F*. *hepatica* metacercariae were provided by Ridgeway Research Ltd (Gloucestershire, UK) and stored at 4°C on 0.4% carboxymethylcellulose until use. Metacercariae viability was confirmed by microscope observation before infection.

A total of 9 BALB/c mice were used in the present study and were randomly allocated into three experimental groups. Six mice were each orally infected with a volume of 30 μL of PBS solution containing five *F*. *hepatica* metacercariae and served as experimental groups. Three additional mice were used as uninfected controls and were orally administered with a volume of 30 μL of PBS. The ssize.fdr package for R was used to estimate the sample size, taking into account the recommendations by Orr MP *et al*., 2009 [20].

Mice were humanely euthanized with an intraperitoneal injection of pentobarbital (60mg/kg), according to standardized protocols supplied by the University of Salamanca’s Ethics Committee, at the beginning of the experiment (n = 3; uninfected controls; t0), at 7 days post infection (p.i) (n = 3; t7) and at 21 days p.i. (n = 3; t21). Procedures were carried out at University of Salamanca’s Animal Experimentation Facility laboratories. To assess the success of the infection, an ELISA assay was carried out to detect specific antibodies raised against the excretory/secretory antigen from *F*. *hepatica* at the time of necropsy. The physical condition of animals was daily monitored (twice a day) by our trained and qualified personnel on handling of animals used for experimentation.

The livers from each mouse were collected, visually examined and then preserved in either formalin or RNAlater solution for histological analysis and RNA isolation, respectively.

### Histological Assessment

Representative samples were collected from the right and left liver lobes from each mouse to perform the histopathological studies, and both macroscopic and microscopic lesions were examined. These samples were fixed in 10% neutral-buffered formalin and embedded in paraffin wax. Sections (4 μm) were stained with hematoxylin and eosin to determine the liver damage areas.

### RNA isolation, cDNA synthesis and array hybridization

Total RNA was isolated from liver tissues (40 mg) using the Gen Elute Mammalian Total RNA Miniprep Kit (Sigma, San Louis, USA), according to the manufacturer’s instructions. The RNA concentration was quantified using a NanoDrop ND-1000 spectrophotometer (NanoDrop Technologies), and the quality of each sample was assessed using an Agilent 2100 Bioanalyzer (Agilent Tech, Palo Alto, CA, USA) on the basis of the RNA integrity number (RIN). The samples selected for further analysis had integrity factors (RIN) in the range of 7.3–10. Therefore, a total of nine individual liver RNA samples were used for cDNA synthesis and microarray analysis. Labeling and hybridizations were performed according to protocols from Affymetrix. Briefly, 100–300 ng of total RNA was amplified and labeled using the Ambion WT Expression Kit for Affymetrix GeneChip (Invitrogen) and then hybridized to the GeneChip Mouse Gene 1.0 ST Array (Lot. No. 4138271; Affymetrix). Washing and scanning were performed using the GeneChip Instrument System of Affymetrix (GeneChip Hybridization Oven 640, GeneChip Fluidics Station 450 and GeneChip Scanner 3000 7G). The 169-format GeneChip Mouse Gene 1.0 ST Array contained approximately 25 probes that were designed across the full length of 28,853 well-annotated genes for mouse. The design of the array was based on the February 2006 mouse genome sequence (UCSC mm8, NCBI build 36) with comprehensive coverage of RefSeq (http://www.affymetrix.com/support), putative complete CDS GenBank transcripts, all Ensembl transcript classes and synthetically mapped full-length mRNAs and RefSeq NMs from humans and rats. The GeneChip Mouse Gene 1.0 ST Array has 100 percent coverage of NM sequences present in the April 3, 2007 RefSeq database (http://www.affymetrix.com/support).

### Microarray data analysis

After hybridization, scanning, and image acquisition, the data extracted from the raw images were analysed using the Expression Console software (Affymetrix), which enables probe set summarization as well as preliminary data quality evaluation. The resulting data (.CEL files) were further analyzed using the Babelomics V4.2 platform [21], which is freely accessible at http://babelomics.bioinfo.cipf.es/. For data normalization and preprocessing, the Affymetrix raw data files (.CEL files) were background-adjusted, normalized and log-transformed of the perfect match (PM) values using a robust multi-array average (RMA) methodology [22]. The intensity signal was standardized across arrays via the quantile normalization algorithm [23].

A comparison in the gene expression profile at each experimental time point was investigated, thus the following comparisons were performed: t0 *vs* t7, t0 *vs* t21 and t7 *vs* t21. It is worth to note that in the present study we do not provide the gene expression profile values at any individual time but we reported comparisons amongst the different times of sampling. Differential gene expression assessment of all comparisons was performed using Limma moderated t-statistics [24], reporting a t-test statistic for each gene together with its corresponding p-value (generally called raw p-values). These raw p-values were further corrected for multiple testing to minimize the number of *false positives* in the study. In this analysis, we used the conventional multiple testing p-value correction procedures proposed by Benjamini & Hochberg [25] to derive adjusted p-values. For functional profiling, each of the comparisons used here (t0 *vs* t7; t0 *vs* t21 and t7 *vs* t21) were analysed using logistic regression models to identify the functional blocks that were enriched in any of the conditions [26, 27].

Finally, the functional blocks from two databases, GO Biological Process (http://www.geneontology.org) and KEGG Pathways (http://www.genome.jp/kegg/), were used in the study. The Ingenuity Pathways Analysis (IPA) software v6.0 (Ingenuity Systems, www.ingenuity.com) was used to organize the genes that were regulated during the infection into networks of interacting molecules. The gene identifiers of the genes with a statistically significant change in expression (*p*-value < 0.05) and with a calculated positive or negative fold change of at least two-fold were uploaded in the software. These genes, called focus genes, were overlaid onto a global molecular network that was developed from the information contained in Ingenuity’s Knowledge Base. The networks were then algorithmically generated based on their connectivity. Each network is assigned a score, a numerical value that ranks the networks according to how relevant they are to the genes in the uploaded dataset, which is determined according to the number of focus genes and size of the network. In addition, IPA was used for a functional analysis to identify the biological functions that were most significant to the uploaded datasets. The right tailed Fisher’s exact test was used to calculate a p-value for the probability that each biological function assigned to that dataset is due to chance alone.

### PCR

RNA from each of the biological samples was reserved for reverse-transcription PCR to validate a subset of the microarray data. Total RNA from each mouse liver sample was reverse transcribed using the 1st Strand cDNA Synthesis Kit for RT-PCR (AMV) (Cat. no. 1148318801, Roche) in a volume of 20 μL according to the manufacturer’s instructions. The cDNA concentration was measured using a NanoDrop-ND1000 (NanoDrop Technologies). The genes and primers used for PCR were representative of the transcripts that were significantly up- or down-regulated during microarray analysis, and they were designed using Primer-Blast software (http://www.ncbi.nlm.nih.gov/tools/primer-blast) (S1 Table). A touchdown PCR (TD-PCR) was performed with an initial denaturation at 94°C for 1 min, which was followed by a touchdown program for 16 cycles with successive annealing temperature decrements of 1.0°C every 2 cycles. For these 2 cycles, the reaction was denatured at 94°C for 20 s, which was followed by annealing at 61°C-54°C for 30 s and polymerization at 72°C for 30 s. The subsequent 15 cycles of amplification were similar, except that the annealing temperature was 53°C. The final extension was performed at 72°C for 10 min. The TD-PCR amplification was performed in a Mastercycler gradient (Eppendorf) and confirmed by electrophoresis. Genomic DNA contamination was ruled out for each RNA sample by amplification of each RNA sample without previous reverse-transcription step.

## Results

### Experimental infection

To assess the success of the experimental infection, an ELISA assay was carried out at the time of necropsy. As expected, absence of IgG antibodies was detected in 3/3 mice belonging to uninfected-control groups. At 7 days p.i, we detected low IgG levels in 3/3 mice, and at 21 days p.i we detected high IgG levels in 3/3 mice (Data not shown). All mice used in this study (9/9) remained alive during the whole time-course experiment. According to our health surveillance program according to Federation of European Laboratory Animal Science Associations (FELASA) guidelines, the status health of all animals used here was optimal during the experimentation phase. We do not evidence any symptoms of severe pain, excessive distress, suffering or an impeding death in any of the animals.

### Histological analysis

**Fig 1:**
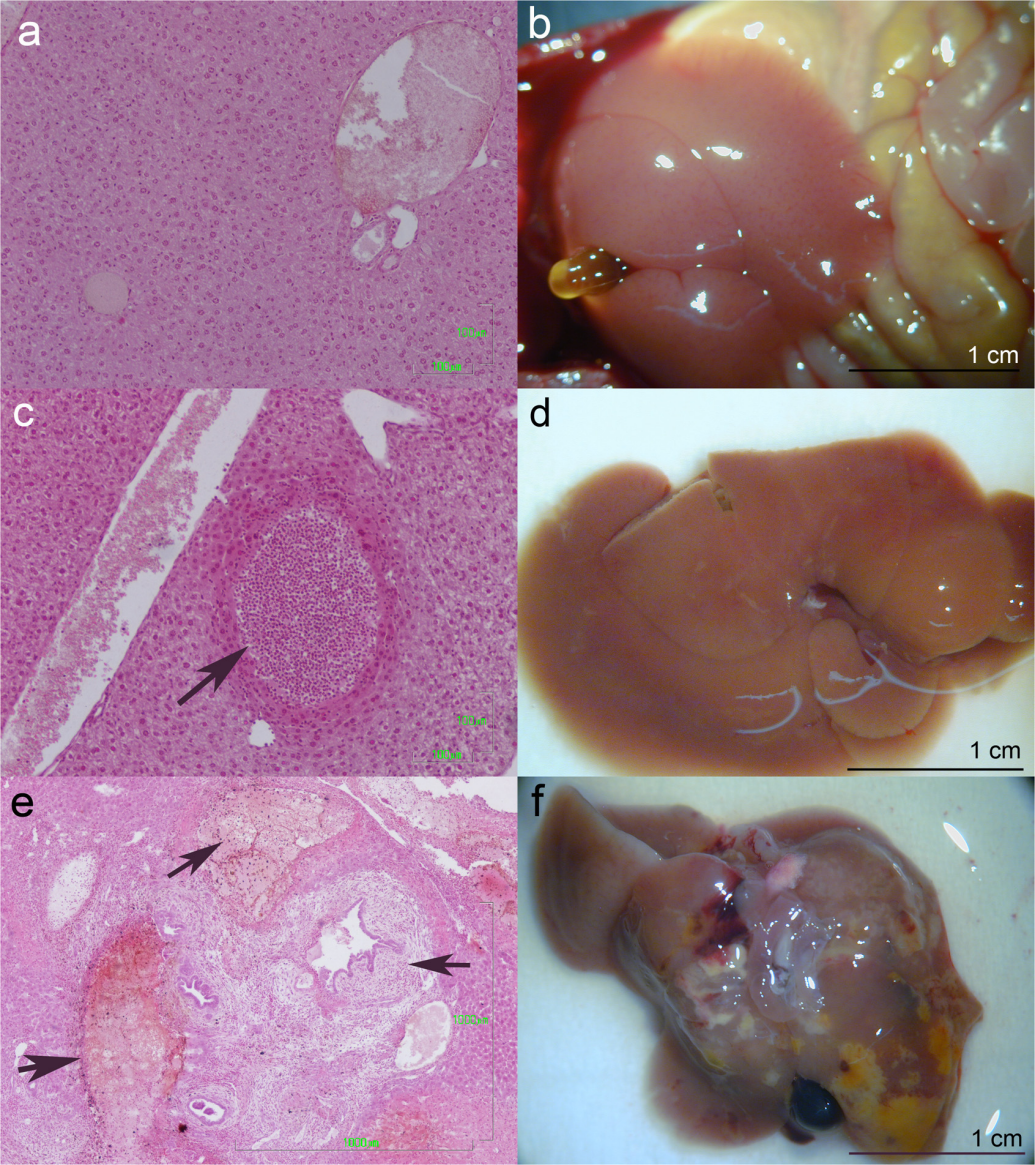
Macro and microscopic hepatic lesion of mice infected orally with 7 metacercariae of *F*. *hepatica*. (A, B) Uninfected control. Micrography section of a migration tract with infiltrates (C) and the liver with whitish tracts and spots in the parenchyma (D) at 7 days p.i (t7). Liver parenchyma with extensive areas of necrosis, fibrosis, cholangitis and granulomas with infiltrates (E) and severe macroscopic damage (F) at 21 days p.i (t21).

The livers from mice belonging to each experimental group were histologically assessed to compare the hepatic damage progress during the experimental infection. At 7 days p.i, we observed thin whitish tracts and spots in the liver parenchyma. A microscopic analysis revealed abundant infiltrates of neutrophils, eosinophils and macrophages in the section of migration tracts, which can be attributed to the wandering of immature flukes. At 21 days p.i, we observed severe liver tissue damage, bile duct enlargement, superficial scars, and irregular yellowish-white areas on the liver surface and into the parenchyma. A microscopic analysis revealed extensive areas of necrosis, fibrosis, cholangitis and granulomas in the liver parenchyma with diffuse infiltration of eosinophils, macrophages, lymphocytes and plasma cells (Fig 1).

### Data normalization and pre-processing

The data discussed in this publication have been deposited in NCBI’s Gene Expression Omnibus [28] and are accessible through GEO Series accession number GSE69588 (http://www.ncbi.nlm.nih.gov/geo/query/acc.cgi?acc=GSE69588). All samples could be read and pre-processed appropriately. The box plots showing intensity distribution for each of the arrays in this study and data distributions before and after normalization are shown in S1 Fig. The normalized data for each of the probe-sets on the GeneChip Mouse Gene 1.0 ST Array were filtered for significant signal and normalized to uninfected controls, reducing the data set to 28,853 genes, of which, 5,631 were shown to be differentially expressed. Genes that were differentially expressed were chosen according to the following criteria: *i)* at least ± 2-fold change in their expression compared to the uninfected control group and *ii)* a statistic *p* value < 0.05.

### *Fasciola hepatica* infection induces changes in the gene expression in the liver

In the present study, we investigated the gene expression profile in the livers of mice from infection with *F*. *hepatica* metacercariae at 7- and 21-days post-infection, which corresponds to parasite migration through the hepatic parenchyma and subsequent establishment of mature adults in the liver, respectively.

**Fig 2:**
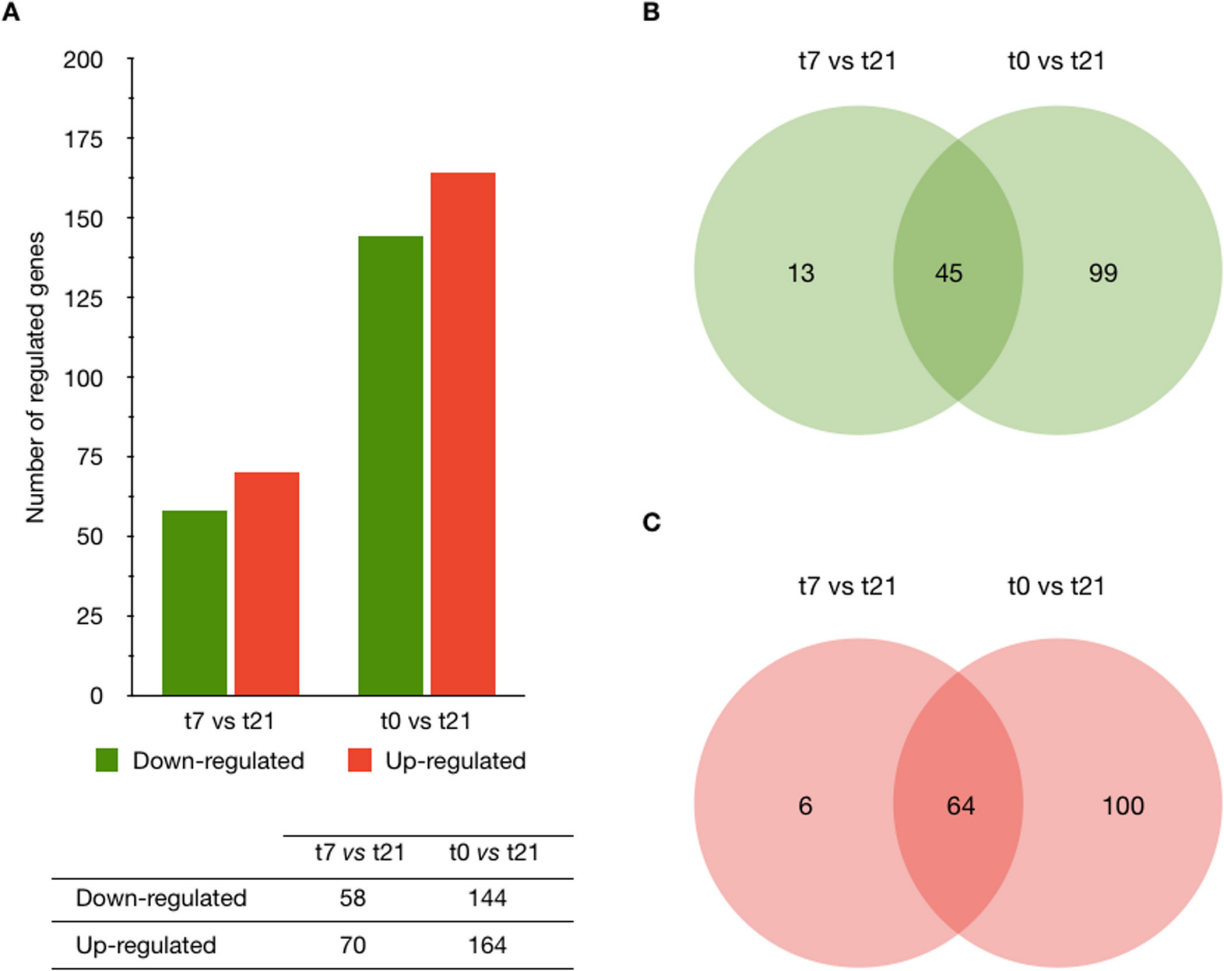
The number of genes differentially expressed as a result of infection with *F*. *hepatica* metacercariae. (A) A Venn diagram representing the differentially expressed genes obtained from statistical analyses of two groups (t7 *vs* t21 and t0 *vs* t21; Significant Analysis of Microarrays SAM test, false discovery rate at 5%). The figure represents the numbers of probe sets that are differently expressed between the three groups (untreated controls t0, infected and sacrificed at 7 days p.i (t7), and infected and sacrificed at 21 days p.i (t21)) and shared among the analyses (t7 *vs* t21, and t0 *vs* t21). (B) Down-regulated genes. (C) Up-regulated genes. Down-regulated genes are represented as green bars and up-regulated genes as red bars.

Interestingly, only 5 genes were differentially expressed (*p* < 0.05) when comparing t0 *vs* t7 and none of those genes presented a fold change value equal or higher than ± 2. Comparison between t7 *vs* t21 allowed for the identification of 1,993 genes that were differentially expressed (*p* < 0.05). However only 128 of those genes presented a fold change value equal or higher than ± 2; 58 of them being down-regulated and 70 being up regulated. Similarly, we identified 3,633 genes that were differentially expressed (*p* < 0.05) when a comparison between t0 *vs* t21 was performed. Only 436 of those genes presented a fold change value equal or higher than ± 2; 202 of them were down-regulated and 234 up-regulated (Fig 2A).

Intersection unit tests (IUTs) using Venn diagrams show the numbers of genes that are differentially expressed at different times of infection (t7 *vs* t21 and t0 *vs* t21), as can be seen in Fig 2B and 2C. It is worth noting that *F*. *hepatica* infection mostly induces up-regulation of genes at any time of infection. A complete list of all the genes that are differentially expressed in both early and late infection is given in S2 Table.

### Gene ontology (GO) and pathway analysis

**Fig 3:**
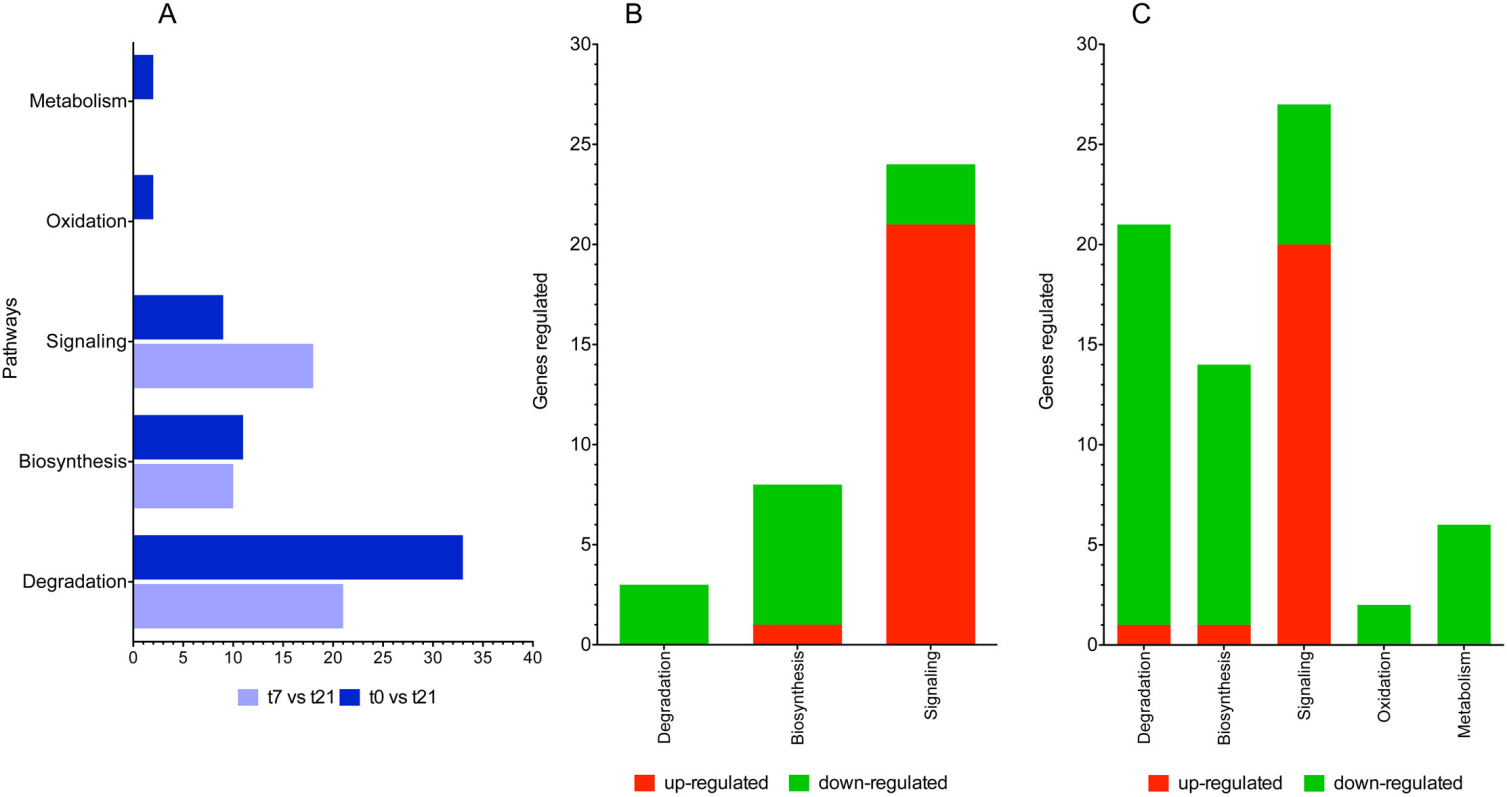
Representative canonical pathways study using the ingenuity pathway analysis (IPA) tool. (A) A comparison of the numbers of pathways that are statistically significant is shown for each point (t7 vs t21 and t0 vs t21). (B) The numbers of genes that are differentially expressed in each of the pathways are depicted for t7 vs t21 and (C) t0 vs t21. Down-regulation is shown in green and up-regulation in red.

The GO and pathway analysis was performed using Babelomics V4.2 and Ingenuity Pathway Analysis, making comparisons between infection at t7 and t21 to the uninfected control group. In both cases (comparisons between t7 *vs* t21 and t0 *vs* t21), we found the following pattern concerning the number of genes being differentially expressed in canonical pathways: degradation > biosynthesis > signaling. Comparison between t7 *vs* t21 revealed 64 canonical pathways with a *p*-value < 0.05, whereas comparison between t0 *vs* t21 revealed 69. Comparison between t0 *vs* t21 also showed an increase in the number of pathways that are associated with degradation and a reduction in the signaling-associated pathways (Fig 3A). Strong down-regulation was observed in both cases in pathways associated with degradation and biosynthesis, whereas up-regulation was achieved in the signaling pathways (Fig 3B and 3C). The pathways associated with oxidation and metabolism was only present in the comparison between t0 *vs* t21, which was accompanied by a strong down-regulation (Fig 3C). The number of genes that were differentially expressed (both up- and down-regulated) is greatest at t21.

**Fig 4:**
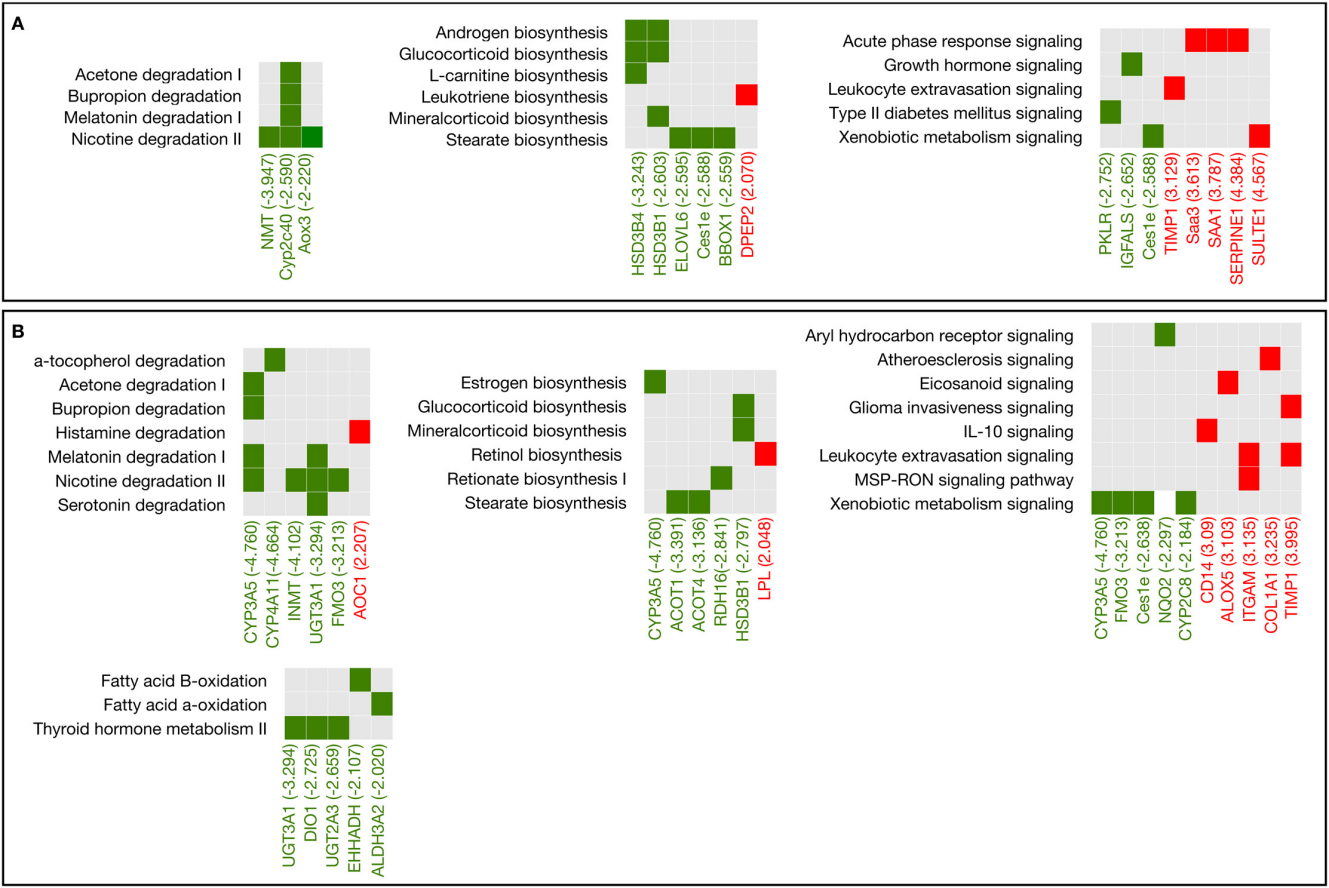
The most representative pathways and the associated differentially expressed genes in two stages of the infection. (A) Comparison between t7 (seven days post-infection) vs t21 (21 days post-infection). (B). Comparison between t0 (Uninfected) vs t21 (21 days post-infection). The fold change of each gene is shown in parentheses. Red squares represent the genes that are up-regulated, belonging to each signaling pathway, green squares represent genes that are down-regulated, whereas gray squares indicate the absence of those genes in that pathway.

At this stage of infection, *F*. *hepatica* induces the alteration of a greater number of pathways as well as the differential expression of a greater number of genes. It is evident that strong down-regulation is achieved during *F*. *hepatica* infection in terms of the degradation, biosynthesis, oxidation and metabolism pathways in both early and late infection (Fig 4A). However, strong up-regulation is observed in pathways related to signaling in both stages of infection (Fig 4B).

Concerning the degradation and biosynthesis pathways, we observed a strong down-regulation of several genes that belong to the cytochrome P450 (e.g., CYP3A5, Cyp2c40, CYP4F12, and CYP2C8), glucoronosyltransferases (e.g., UGT3A1, UGT2A3, UGT2B17, UGT2B28), carboxylesterase (Ces1e), fatty-acid elongase (ELOVL), aldehyde dehydrogenase (ALDH) and other degradation-associated genes, such as arylformamidase (AFMID) and aldehyde oxidase (Aox3). Analysis of the signaling pathways also revealed up-regulation of many genes, including the chemokine C-X-C motif receptor 4 (CXCR4), tumor necrosis factor receptor family (TNFRSF), suppressor of cytokine signaling (SOCS), CD14 molecule (CD14) and metalloprotease inhibitor (TIMO), amongst others. Table 1 shows the significant genes (down- and up-regulated) that are associated with the canonical pathways related to degradation, biosynthesis and signaling at 21 days after the infection.

**Table 1:** Genes associated with pathways related to degradation, biosynthesis and signaling.

Pathway/Gene symbol	Gene description	Fold change	p-value
**Degradation**			
CYP3A5	Cytochrome P450, family 3, subfamily A, polypeptide 5	-4.760	1.52E-03
CYP4A11	Cytochrome P450, family 4, subfamily A, polypeptide 11	-4.664	8.20E-04
INMT	Indolethylenamine N-methyltransferase	-4.100	2.28E-04
UGT3A1	UDP glucoronosyltransferase 3 family, polypeptide A1	-3.294	2.79E-04
FMO3	Flavin containing monooxygenase 3	-3.213	1.22E-04
AFMID	Arylformamidase	-2.749	7.45E-06
UGT2A3	UDP glucoronosyltransferase 2 family, polypeptide A3	-2.659	1.99E-04
Cyp2c40	Cytochrome P450, family 1, subfamily c, polypeptide 40	-2.469	9.51E-05
UGT2B17	UDP glucoronosyltransferase 2 family, polypeptide B17	-2.438	1.44E-03
LIPG	Lipase, endothelial	-2.434	2.33E-04
CYP4F12	Cytochrome P450, family 4, subfamily F, polypeptide 12	-2.340	2.92E-04
MGLL	Monoglyceride lipase	-2.314	1.07E-06
SULT1B1	Sulfurotransferase family, cytosolic 1B, member 1	-2.284	2.88E-04
CYP2C8	Cytochrome P450, family 2, subfamily C, polypeptide 8	-2.184	1.43E-03
MAOB	Monoamine oxidase B	-2.174	1.11E-04
Aox3	Aldehyde oxidase 3	-2.120	3.38E-05
EHHADH	Enoyl-CoA, hydratase/3-hydroxyacyl CoA	-2.107	1.69E-04
ALDH3A2	Aldehyde dehydrogenase 3 family, member a2	-2.020	3.76E-08
UGT2B28	UDP glucoronosyltransferase 2 family, polypeptide B28	-2.007	2.38E-04
**Biosynthesis**			
CYP3A5	Cytochrome P450, family 3, subfamily A, polypeptide 5	-4.760	1.52E-03
ACOT1	Acyl-CoA thioesterase 1	-3.391	1.01E-04
ACOT4 RDH16	Acyl-CoA thioesterase 4 Retinol dehydrogenase 16	-3.136–2.841	7.33E-06 5.52E-05
HSD3B1	Hydroxy-delta-5-steroid dehydrogenase, 3 beta-isomerase 1	-2.797	1.03E-03
AFMID	Arylformamidase	-2.749	7.45E-06
SDR9C7	Short chain dehydrogenase/reductase family 9C, member 7	-2.639	2.88E-04
Ces1e	Carboxylesterase 1E	-2.638	1.62E-04
BBOX1	Butyrobetaine, 2-oxoglutarate dioxygenase	-2.635	1.66E-04
Cyp2c40	Cytochrome P450, family 1, subfamily c, polypeptide 40	-2.469	9.51E-05
LIPG	Lipase, endothelial	-2.434	2.33E-04
ELOVL6	ELOVL fatty acid elongase 6	-2.376	8.21E-04
CYP2C8	Cytochrome P450, family 2, subfamily C, polypeptide 8	-2.184	1.43E-03
LPL	Lipoprotein lipase	2.048	2.46E-05
**Signaling**			
CYP3A5	Cytochrome P450, family 3, subfamily A, polypeptide 5	-4.760	1.52E-03
FMO3	Flavin containing monooxygenase 3	-3.213	1.22E-04
Ces1e	Carboxylesterase 1E	-2.638	1.62E-04
NQO2	NAD(P)H dehydrogenase, quinone 2	-2.297	2.33E-06
CYP2C8	Cytochrome P450, family 2, subfamily C, polypeptide 8	-2.184	1.43E-03
ALDH3A2	Aldehyde dehydrogenase 3 family, member a2	-2.020	3.76E-08
LPL	Lipoprotein lipase	2.048	2.46E-05
CXCR4	Chemokine (C-X-C motif) receptor 4	2.052	3.85E-04
NCF1	Neutrophil cytosolic factor 1	2.087	7.51E-05
ACTA2	Actin, alpha 2, smooth muscle, aorta	2.109	5.03E-04
TNFRSF12A	Tumor necrosis factor receptor superfamily, member 12A	2.122	3.49E-04
MYC	v-myc avian myelocytomatosis viral oncogene homolog	2.180	1.61E-04
SOCS3	Suppressor of cytokine signaling 3	2.199	9.27E-08
RPL13A	Ribosomal protein L13a	2.253	1.32E-05
SELP	Selectin P	2.266	1.21E-03
COL3A1	Collagen, type III, alpha 1	2.347	9.96E-06
FOS	FBJ murine osteosarcoma viral oncogene homolog	2.553	5.57E-04
ALOX15	Arachidonate 15-lipoxygenase	2.562	3.18E-04
COL1A2	Collagen, type I, alpha 2	2.605	5.71E-05
IL1R2	Interleukin 1 receptor, type II	2.692	2.64E-04
HMOX1	Heme oxygenase 1	2.816	4.24E-06
CD14	CD14 molecule	3.094	1.19E-04
ALOX5AP	Arachidonate 5-lipoxygenase-activating protein	3.103	1.02E-05
ITGAM	Integrin, alpha M	3.135	1.24E-04
COL1A1	Collagen, type I, alpha 1	3.235	7.40E-06
TIMP1	TIMO metalloprotease inhibitor 1	3.995	7.07E-05

Only those genes with both a p-value < 0.05 and a fold change ± 2 are included. Genes in this analysis belong to the comparison between t0 (uninfected mice) and t21 (mice infected and necropsied at 21 days after infection).

### Identifying genes involved in causing hepatic toxicity

**Fig 5:**
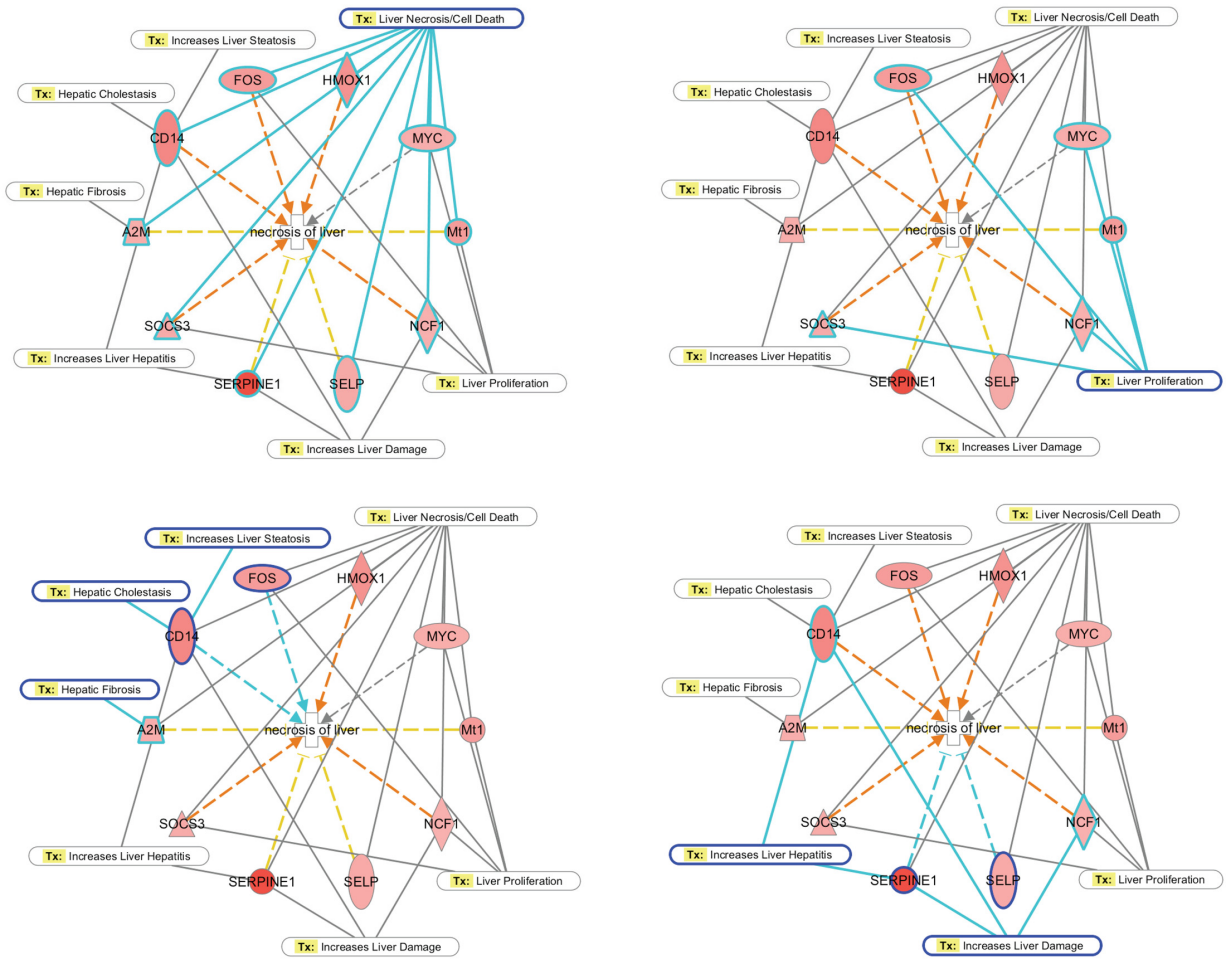
Gene networks associated with hepatic damage. The Ingenuity Pathway Analysis tool was used to identify significant genes that are related to hepatic damage as well as to build up interaction networks amongst them with the most significant associated-functions related to hepatic damage. Solid lines denote direct interactions and dotted-arrows predict activation pathways. The color red indicates up-regulation. The data used for such network construction were the comparison between t0 (uninfected) and t21 (21 days post-infection), which is where the most significant differential expression of genes occurs.

The Ingenuity Pathway Analysis tool, "Tox functions", was used to identify a subset of genes and biological functions that are implicated in causing hepatic toxicity. We identified a strong up-regulation of several genes that promote hepatic toxicity in both stages of infection (t7 *vs* t21 and t0 *vs* t21). In the first stage of infection, five genes that are associated to liver damage, liver necrosis and liver proliferation were identified. Down-regulation was completely absent at this point of infection (Table 2). At 21 days post-infection, we identified a total of 11 regulated genes that were associated with liver damage, liver necrosis and liver fibrosis, and most of them are up-regulated. Strikingly, down-regulation was observed in only 1 gene at this stage of infection (Table 2). We also used the build-up networks and pathways from the Ingenuity Pathway Analysis tool to investigate the relationships between the most significant associated-functions related to the hepatic damage and those individual genes involved in causing liver necrosis and the death of hepatic cells. As in Fig 5, there is a complex interaction, which mainly involves up-regulated genes. Such up-regulation directly induces the activation pathways that lead to liver necrosis, which is mainly through the genes SERPINE1, CD14, FOS, HMOX1, MYC, Mt1, SOCS3, A2M and SELP. Beyond the active participation of these genes in causing liver necrosis, they are also involved in the interaction network, causing hepatic cell death, liver proliferation, increased liver damage, liver hepatitis, hepatic fibrosis, hepatic cholestasis and liver steatosis.

**Table 2:** Biological function and genes involved in causing hepatic toxicity followed by *Fasciola hepatica* infection assessed using the Tox Function from the Ingenuity Pathway Analysis tool.

Gene symbol	Gene description	Fold change	Biological function	p-value
**t7 vs t21**				
BCL2L1	BCL2-like 1	6.326	Liver damage	1.64E-02
			Liver injury	2.23E-02
			Liver necrosis	2.37E-02
CD14	CD14 molecule	3.119	Liver necrosis	2.37E-02
CXCR4	Chemokine (C-X-C motif) receptor 4	2.141	Liver proliferation	2.08E-02
SERPINE1	Serpin peptidase inhibitor, clade E, member 1	4.348	Liver damage	1.64E-02
			Liver injury	2.23E-02
			Liver necrosis	2.37E-02
TNFRSF12A	Tumor necrosis factor receptor superfamily, member 12A	2.175	Hepatocyte expansion	1.54E-03
			Oval cell proliferation	2.08E-02
			Oval cell expansion	3.93E-02
**t0 vs t21**				
SERPINE1	Serpin peptidase inhibitor, clade E, member 1	4.348	Liver damage	4.88E-03
CD14	CD14 molecule	3.119	Liver necrosis	2.58E-03
LGALS3	Lectin, galactoside-binding, soluble, 3	2.898	Liver damage	4.88E-03
FOS	FBJ murine osteosarcoma viral oncogene homolog	2.553	Liver necrosis	2.58E-03
Mt1	Metallothionein 1	2.550	Liver damage	4.88E-03
			Liver necrosis	2.58E-03
Ccl2	Chemokine (C-C motif) ligand 2	2.470	Liver damage	4.88E-03
SELP	Selectin P	2.266	Liver necrosis	2.58E-03
SPP1	Secreted phosphoprotein 1	2.257	Hepatic stellate cell migration	2.48E-02
MYC	v-myc avian myelocytomatosis viral oncogene homolog	2.180	Liver necrosis	2.58E-03
TNFRSF12A	Tumor necrosis factor receptor superfamily, member 12A	2.122	Hepatocyte expansion	9.75E-03
NCF1	Neutrophil cytosolic factor 1	2.087	Liver damage	4.88E-03
			Hepatic stellate cell migration	2.48E-02
			Liver necrosis	2.58E-03
PPARA	Peroxisome proliferator-activated receptor alpha	-2.001	Liver damage	4.88E-03
			Hepatocyte damage	7.94E-03
			Liver necrosis	2.58E-03

The fold change of each gene and significance of each biological function are also shown. The following comparisons were made: *i)*. t7 (mice infected and necropsied at 7 days post-infection) vs t21 (mice infected and necropsied at 21 days post-infection) and *ii)*. t0 (uninfected mice) vs t21 (mice infected and necropsied at 21 days post-infection).

### Up-regulation determines hepatic injury in *F*. *hepatica* infection

Chronic *F*. *hepatica* infection causes severe liver damage, including hepatic abscesses, hepatic necrosis and the subsequent destruction of the hepatic parenchyma, leading to clinical manifestations, such as anemia, abdominal disorders and hepatic dysfunction. Here, we identified 20 regulated-genes as the most relevant genes that are associated with such damage; 13 of them are up-regulated and 7 are down-regulated. The aforementioned regulated-genes were then classified into subsets according to their functionality, including liver necrosis, liver damage, fatty acid oxidation and metabolism, among others. Ten genes were functionally assigned as necrosis and liver damage genes; most of those genes (9 of them) were up-regulated, and only 1 was down-regulated (Table 3). In addition to the biological functions associated with liver damage, we also identified other genes that are related to fatty acid oxidation and metabolism (e.g., Peroxisome Proliferative Activated Receptor, Alpha (PPARA) and Solute Carrier Family 22 (Organic Cation/Carnitine Transporter), Member 5 (SLC22A5)), lipid and vitamin metabolism and small molecule biochemistry (e.g., Solute Carrier Family 23 (Ascorbic Acid Transporter), Member 1 (SLC23A1), Retinol Dehydrogenase 16 (RDH16), and ATP-binding cassette, sub-family G, member 8 (ABCG8)).

**Table 3:** Genes involved in causing damage and liver necrosis according to the Ingenuity Pathway Analysis tool.

Biological function/ Gene symbol	Gene description	Fold change	p-value
**Liver damage**			
SERPINE1	Serpin peptidase inhibitor, clade E, member 1	4.348	5.40E-05
LGALS3	Lectin, galactoside-binding, soluble, 3	2.898	3.88E-05
Mt1	Metallothionein 1	2.550	9.11E-04
Ccl2	Chemokine (C-C motif) ligand 2	2.470	1.38E-03
NCF1	Neutrophil cytosolic factor 1	2.087	7.51E-05
PPARA	Peroxisome proliferator-activated receptor alpha	-2.001	2.99E-04
**Liver necrosis**			
SERPINE1	Serpin peptidase inhibitor, clade E, member 1	4.348	5.40E-05
CD14	CD14 molecule	3.119	1.19E-04
FOS	FBJ murine osteosarcoma viral oncogene homolog	2.553	5.57E-04
Mt1	Metallothionein 1	2.550	9.11E-04
SELP	Selectin P	2.266	1.21E-03
MYC	v-myc avian myelocytomatosis viral oncogene homolog	2.180	1.61E-04
NCF1	Neutrophil cytosolic factor 1	2.087	7.51E-05
PPARA	Peroxisome proliferator-activated receptor alpha	-2.001	2.99E-04
**Fatty acid oxidation**			
SLC22A5	Solute carrier family 22, member A5	-2.224	6.69E-04
SLC25A3	Solute carrier family 25A, member 23	-2.158	5.15E-04
PPARA	Peroxisome proliferator-activated receptor alpha	-2.001	2.99E-04

The fold change and significance of each gene are also shown. Regulated genes correspond to the comparison between t0 (uninfected mice) *vs* t21 (mice infected and necropsied at 21 days post-infection).

### PCR for microarray validation

**Fig 6:**
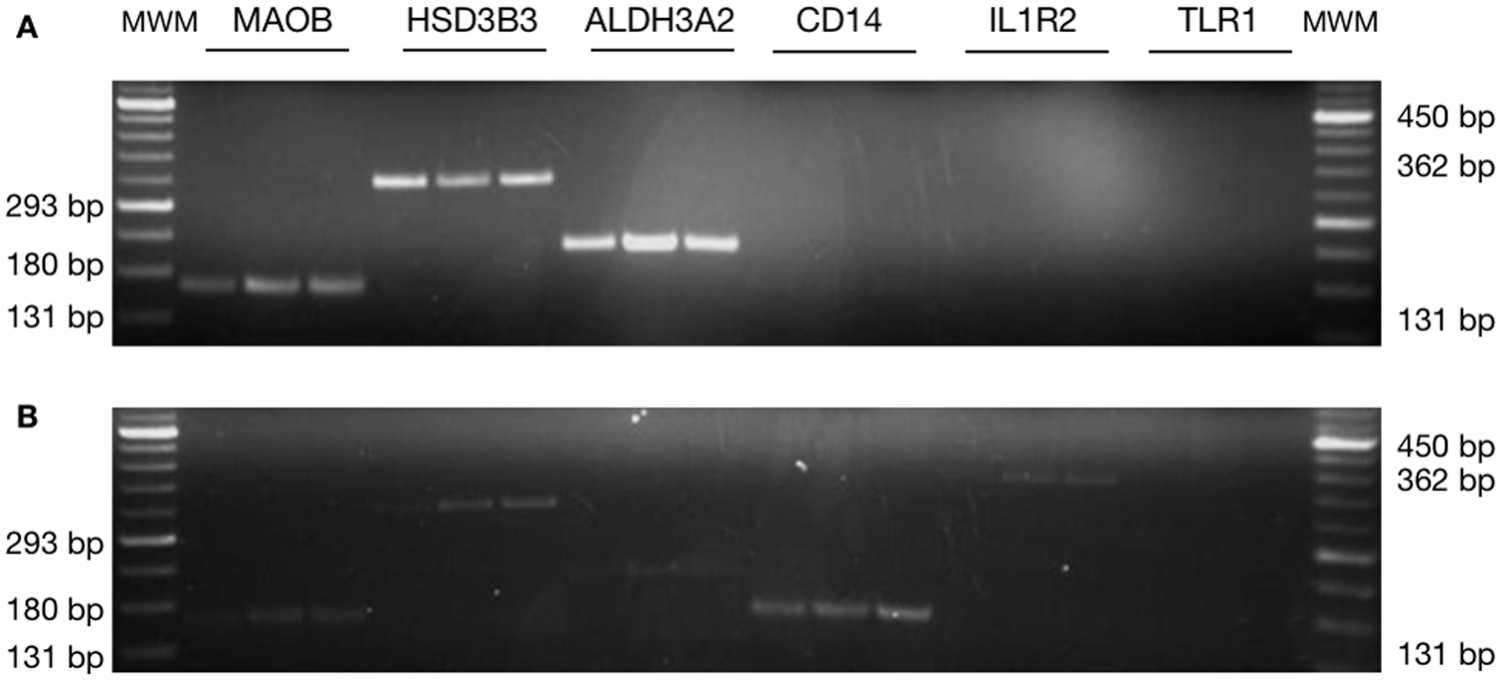
The microarray was validated with PCR reactions. (A) PCR amplification from liver RNA samples for the selected genes at time t0 (before any treatment). (B) PCR amplification at t21 (21 days after oral infection of mice with *F*. *hepatica* metacercariae). Three up-regulated genes and three down-regulated genes were randomly chosen for PCR amplification. The corresponding PCR for each gene was performed using each biological sample obtained at t0 and t21. The results are representative of three individual experiments. MWM: molecular weight marker.

The PCR expression patterns agreed with those obtained in the microarrays. Six genes were randomly selected for such confirmation (three up-regulated genes and three down-regulated ones). As in Fig 6, all genes that were up-regulated had a higher band intensity in the group of mice infected with *F*. *hepatica* metacercariae compared to the uninfected group, whereas the down-regulated genes had lower band intensity in the infected group.

## Discussion

In the present study, we used microarray-based methodology to assess the gene expression profile in the livers of mice infected with *F*. *hepatica* metacercariae, which was combined with histological analysis to identify the differential expression of the genes that are associated with inducing liver damage as well as to evaluate the hepatic damage during the infection. With respect to gross examination of the liver, we observed enlargement together with hemorrhages on the liver surface, which were mainly associated with inflammatory reactions and fibrosis, and two-thirds of the hepatic tissue was affected. We also observed areas with a high density of lymphocytes, which were mainly marked by the migration of juvenile flukes. These anomalies have also been observed in other fasciolosis models, and the highest damage takes place in the chronic phase of the disease [9, 29]. On the other hand, the infection caused by *F*. *hepatica* also induced significant changes in the gene expression profile in the livers of affected mice compared with the untreated control group. Out of 28,853 probe-genes in the array, the expression of 3,775 (13.1%) was changed at a *p*-value < 0.05, and 327 of them (8.7%) at a fold change equal to ± 2. The immune response induced by *F*. *hepatica* has been well studied in different experimental models, resulting in a highly Th2-dependent response with an increase in not only the number of eosinophils and antibodies, such as IgG1 and IgE, but also in the Th2-cytokine associated profile, including IL-4, IL-5 and IL-10 [30]. Concerning immune response, in the present study we identified the differential expression of several relevant genes associated with such immune response induced by *F*. *hepatica*. We found up-regulation of genes associated with the innate immune response, such as TLR1, TLR3 and TLR4 and also up-regulation of costimulatory molecules, such as CD81 and CD84 and cytokines such as IL-1 and IL5. These results are in concordance with those obtained by [31], who also investigated the gene expression profile in sheeps experimentally infected with *F*. *hepatica*.

However, little is known about the gene expression profile during *F*. *hepatica* infection, and the genes involved in causing liver injury and the subsequent liver pathology responsible for the clinical manifestations of the disease have not been identified. To date, there are only two reports in the literature about the use of microarray and RNA-seq-based experiments after *F*. *hepatica* infection using the rat and the sheep as infection model [31, 32].

More recently, a draft genome for *F*. *hepatica* has been published and time-course of gene expression was performed to investigate those mechanisms used by *F*. *hepatica* to successfully adapt and survive into the mammalian host by using RNAseq-based methodology [33]. A progressive differential expression of a large number of genes as the parasite grows and migrates into the mammalian host has been found, with predominance of cathepsins which involvement during infection has been widely studied. Another important issue concerning the success of *F*. *hepatica* establishment in the mammalian host is related with a very sophisticated anti-oxidant system essential not only for the detoxification of reactive oxygen and nitrogen species but also as defense against superoxide and nitric oxide produced by cell of the mammalian host. Genes encoding superoxide dismutase (SOD), glutathione transferases (GSTs) and fatty acid binding proteins (FABPs) are within the most important anti-oxidants expressed by *F*. *hepatica*. Such detoxification pathway comprises three main phases; phase I (activation), phase II (conjugation) and phase III (efflux). The last phase has also several implications in reducing anti-helminthic drug activity, bioavailability and has also been linked to triclabendazole resistance [33].

It is well known than the liver is an organ where the biotransformation of xenobiotics and drugs occurs, and it is implicated in a number of biological functions, including carbohydrate metabolism, fatty-acid metabolism, protein metabolism, hormone metabolism and the storage of glycogen and vitamins. The liver also has immunological and defense mechanisms through the action of the Kupffer cells, sinusoidal endothelial cells and natural killer cells [34].

Here, we identified a strong down-regulation in the pathways that are associated with degradation, biosynthesis and signaling from *F*. *hepatica* infection at 21 days after the experimental challenge. Several genes belonging to the cytochrome P450 family (CYP) were found to be highly down-regulated in such pathways, especially the CYP3A5 gene (cytochrome P450, family 3, subfamily A, polypeptide 5).

The study of the CYP450 system has previously been described to understand the resistance/susceptibility to triclabendazole of some *F*. *hepatica* isolates by using *in vitro* experiments, demonstrating that such resistance/susceptibility is altered by the inhibition of triclabendazole metabolism from the addition of some inhibitors, including the CYP450 inhibitor, ketoconazole [35–37]. Further *in vivo* experiments also demonstrated that resistance to triclabendazole was diminished by the alteration in drug metabolism with CYP450 inhibitors [38, 39], confirming the importance of the CYP450 system in *F*. *hepatica* susceptibility/resistance. However, others have reported increased activity of the isoenzyme CYP2A5 in the livers of mice that were experimentally challenged with *F*. *hepatica* and has also been correlated with liver injury, cell death and hepatocyte proliferation [40]. Our study also demonstrated a correlation between the regulation of the CYP3A5 gene and time after infection, and there was more enhanced down-regulation with infection. Other studies have also investigated the participation of the CYP450 family enzymes as mediators in the formation of oxysterols, products of cholesterol oxidation that could induce cholangiocarcinogenesis in the infection caused by the liver fluke *Opisthorchis viverrini* [41–43]. Oxysterols have also been associated with the development of other pathologies and various cancer types [44–46]. Induction of cholangiocarcinogenesis by *O*. *viverrini* has also been associated with a down-regulation of galectin-3, a B-galactoside binding lectin [47]. By contrast, we found up-regulation of the LGALS3 gene, which correlates with the progression of the *F*. *hepatica* infection. We do not find any correlation between the regulation of the CYP family genes, oxysterols and *F*. *hepatica* infection. Reports concerning oxysterols and *F*. *hepatica* are also scarce, which is in accordance with the absence of cholangiocarcinogenesis caused by the liver fluke *F*. *hepatica*.

In our study, we identified a marked up-regulation of several genes that are associated with liver injury, which increases as the infection progresses. Up-regulation of those genes is ranked as follows: SERPINE1 > CD14 > FOS > HMOX1 > MYC > Mt1 > SOCS3 > A2M > SELP > LGALS3 > CCL2 > MCF1 > PPARA. To date, available reports concerning the regulation of those genes and the precise role leading to liver damage in parasite-associated infections are very limited. SERPINE1 (serpin peptidase inhibitor, clade E) member 1 gene has an important role in liver function because it is a potent inhibitor of tissue plasminogen activator (tPA), leading to the inhibition of fibrinolysis, a natural process that causes the formation of blood clots [48]. The most relevant biological pathways associated with the SERPINE1 gene are hepatic fibrosis, hepatic stellate cell activation and the coagulation system.

In summary, we have identified, for the first time, the gene expression pattern induced for *F*. *hepatica* infection using a microarray-based methodology, leading to the identification of those genes that are associated with liver injury and hepatic toxicity as well as those implicated in the degradation, metabolism, signaling and oxidation pathways. The results obtained here could provide information and insights about the molecular pathways in the successful establishment of infection at least in a murine model. Taking into account the results obtained here, they could be useful to study and compare the gene expression profile caused by *F*. *hepatica* in other experimental or naturally-infected models.

The expression pattern obtained here, with CYP3A5 down-regulation expression gene (cytochrome P450 family) and LGALS3 up-regulation expression gene (galectine 3), could also be used to explain the lack of association between infection with *F*. *hepatica* and cholangiocarcinoma. However, more studies are needed to confirm this hypothesis.

## Supporting Information

S1 Fig Raw data file (.CEL files) where background-adjusted, normalized and log-transformed of the perfect match (PM) values using a robust multi-array average methodology (RMA). Box plots showing the global normalized expression values for each RNA sample in non-normalized data (A) and normalized data (B).
(EPS)

S1 File The NC3Rs ARRIVE Guidelines Checklist. (PDF)

S1 Table Specific primer sequence for the PCR amplification of selected genes that are up- and down-regulated. Primers were designed using Primer-Blast software and amplification was conducted by using a touch-down PCR reaction. The free ImageJ software was used or the quantitative digital analysis of image data from electrophoresis gels.
(DOCX)

S2 Table Genes differentially expressed in the livers of mice that are infected with *F*. *hepatica* metacercariae. Comparisons between t0 *vs* t7, t7 *vs* t21 and t0 *vs* t21 were performed. All of the genes with a statistic p-value < 0.05, regardless of its fold change value, are listed in this table.
(XLSX)
