# Supplementary material for: Gene Expression Profile in the Liver of BALB/c Mice Infected with Fasciola hepatica
Source: PLoS One. 2015 Aug 6;10(8):e0134910. doi: 10.1371/journal.pone.0134910 (PMC4527836; doi:10.1371/journal.pone.0134910)
Supplement: S1 Table — Primers were designed using Primer-Blast software and amplification was conducted by using a touch-down PCR reaction. The free ImageJ software was used or the quantitative digital analysis of image data from electrophoresis gels. (DOCX) [file pone.0134910.s003.docx]

**Table 1S**. **Specific primer sequence for PCR amplification of selected genes up- and down-regulated.**

| Gene symbol | Forward  Reverse | Primer length (pb) | Tm (ºC) | GC (%) | Product length  (pb) |
| --- | --- | --- | --- | --- | --- |
| MAOB | TATACTTGGGGACCGAGTGA CAGGTGGAATGGCACTAATC | 20 | 51.24 50.43 | 50.00  50.00 | 131 |
| HSD3B3 | TGGGTATTACCAGTCCAGTA TAAAGCTAGCAGAATTTTCG | 20 | 48.58 45.99 | 45.00  35.00 | 293 |
| ALDH3A2 | ATTGGAGCTTGGAATTACCC GATACCGCCATTAACAATCG | 20 | 49.50 49.08 | 45.00  45.00 | 180 |
| CD14 | TTCCCGACCCTCCAAGTTTT CATCCCGCAGTGAATTGTGA | 20 | 53.19 52.79 | 50.00  50.00 | 130 |
| IL1R2 | CCCGTGCAAAGTGTTTCTGG CCTGCGTTTACACCGTCTGC | 20 | 54.27 56.26 | 55.00  60.00 | 361 |
| TLR1 | TGTGGACACCCCTACAGAAA ATGCTTGAGGCTGACTGTTG | 20 | 52.23 52.69 | 50.00  50.00 | 450 |

Primers were designed using the Primer-Blast software and amplification was conducted by using a touch-down PCR reaction.
